# Small volumes of n-propanol (60%) applied for 3 minutes may be ineffective for surgical hand disinfection

**DOI:** 10.1186/2047-2994-3-15

**Published:** 2014-04-24

**Authors:** Günter Kampf, Christiane Ostermeyer

**Affiliations:** 1 Bode Science Center, Bode Chemie GmbH, Melanchthonstrasse 27, Hamburg 22525, Germany; 2 Institut für Hygiene und Umweltmedizin, Ernst-Moritz-Arndt Universität Greifswald, Walther-Rathenau-Str. 49a, Greifswald 17489, Germany; 3 Microbiology, Bode Chemie GmbH, Melanchthonstrasse 27, Hamburg 22525, Germany

**Keywords:** Surgical hand disinfection, n-propanol, Applied volume, EN 12791, Hand size

## Abstract

**Background:**

There is a trend in some countries to recommend the use of surgical hand disinfectants at volumes as low as 4 ml per application.

**Aim:**

To determine whether the volume applied and hand size influence the efficacy of surgical hand disinfection.

**Methods:**

Thirteen experiments, according to EN 12791, resulting in 269 datasets from 75 subjects were analyzed. Hands were first washed for one minute with soap. The pre-values were obtained by rubbing the finger tips in tryptic soy broth for one minute. Each subject treated his/her hands with n-propanol (60%, v/v), with as many portions as necessary to keep the hands wet for three minutes (6–12 ml). Bacterial post-values were taken from one hand (immediate effect); the other hand was gloved for three hours (sizes 7–9). The second post-value was taken when the glove was removed (3 h effect).

**Results:**

The mean immediate log_10_ reduction of CFU was 2.56 ± 1.12. The glove size had no significant effect on the efficacy of disinfection (p = 0.182; ANOVA). However, a volume of 6 ml was significantly less effective than 9 ml for glove sizes of 7.5–8 (p < 0.05; Tukey post hoc analysis). The mean log_10_ reduction after 3 h was 2.12 ± 1.24. A volume of 6 ml was again significantly less effective than 12 ml for glove size 7 and than 9 ml for glove sizes 7.5–8 (p < 0.05).

**Conclusions:**

The application of small volumes of surgical hand disinfectant when using the EN 12791 reference procedure is likely to yield poor efficacy results, regardless of hand size.

## Background

The application of alcohol-based hand rubs for surgical hand preparation to reduce the risk of surgical site infections is recommended by various organizations, including the World Health Organization (WHO) [1], the Robert Koch Institute (RKI) in Germany [2], and the Centers for Disease Control and Prevention (CDC) in the USA [3]. The efficacy of products is usually determined with national test methods [4] or continental test methods, such as European norms [5]. Label specifications for the correct use of the products are usually based on efficacy data obtained with these test methods. In Europe, the typical mode of application is “thoroughly wet hands and forearms for 1.5 minutes” based on data collected according to EN 12791 [6]. In this situation, a surgeon with large hands may have to use more hand rub than a surgeon with small hands. This variability, of 6–12 ml per application on both hands, has been described before for a 3 min application time [7]. In the USA, it is now recommended that some hand antiseptics be used at volumes as small as 6 ml (application of 3 × 2 ml) [8,9] or even 4 ml (application of 2 × 2 ml) [10] for the entire procedure. In this situation, a surgeon with large hands is encouraged to use the same volume of hand rub as a surgeon with small hands. However, to our knowledge, it has never been demonstrated that the same volume (e.g., 4 ml) of disinfectant is equally effective on large and small hands. We have observed a general trend towards the use of increasingly smaller volumes of hand hygiene products, and not only in surgical hand disinfection [11]. However, this trend may not take into account that larger hands require larger volumes of disinfectant. Therefore, we investigated the efficacy of the reference treatment (EN 12791) in volunteers with different hand sizes who used different amounts of hand rub to keep their hands wet for 3 min.

## Methods

### Test principle and prerequisites

A total of 13 experiments were performed according to EN 12791, including 10 experiments that were published in 2004 [7] and are now reanalyzed in terms of the disinfectant volume and hand size. The bactericidal efficacy of the reference alcohol n-propanol (60%, v/v) *in vivo* was assessed in 18–20 healthy volunteers per experiment [5]. The volunteers were office workers and laboratory staff. No skin breaks, such as cuts or abrasions, or other skin disorders were present. The participants’ nails were short and clean. The volunteers used no substances with antibacterial activity or antibacterial soaps for one week before the tests. Between each experiment, a rest period of at least one week elapsed to allow the reconstitution of the normal skin flora [5]. During that week, antibacterial soaps and hand rubs were avoided. The study was conducted in accordance with the ethical principles that have their origins in the current version of the Declaration of Helsinki (52nd WMA General Assembly, Edinburgh, Scotland, October 2000). Informed consent was obtained from each participant.

### Wash phase

To remove the transient bacterial flora and any foreign particles, the volunteers’ washed their hands with a non-medicated soap (sapo kalinus). A 5 ml sample of the soft soap was poured into the cupped wet hands and rubbed vigorously onto the skin up to the wrists for 1 min, in accordance with the standard procedure, to ensure the total coverage of the hands. The hands were than rinsed with running tap water and dried with a paper towel.

### Determination of the pre-values

The distal phalanges of the right and left hands, including the thumbs, were rubbed separately for 1 min onto two Petri dishes (9 cm diameter), containing 10 ml of tryptic soy broth (TSB), as described in EN 12791 [5]. A 1:10 dilution of the sampling fluid obtained for each hand was prepared in TSB. Aliquots were taken from the sampling fluid (1 ml and 0.1 ml) and the dilution step (0.1 ml) and spread over tryptic soy agar (TSA) dishes with a sterile glass spatula. No more than 30 min elapsed between sampling and seeding. The dishes were incubated for 48 h at 36 ± 1°C and the colony-forming units (CFU) counted when there were between 15 and 300 colonies per plate.

### Disinfection phase

Each of the volunteers applied 60% n-propanol (v/v) for 3 min, as described in the EN 12791 protocol [5], which specifies that the mode of application must be supervised and that each subject must apply as many aliquots of 3 ml as necessary to keep both hands wet for 3 min. The number of 3 ml portions that were required to keep the hands wet with the reference alcohol varied between subjects and was recorded for each application.

### Determination of post-values

After disinfection, the volunteers rubbed the distal phalanges of one hand (randomly selected) in a Petri dish containing 10 ml of TSB supplemented with neutralizers for 1 min (“immediate effect”). The following neutralizers were used: 3% Tween 80, 3% saponin, 0.1% histidine, and 0.1% cysteine [12]. The other hand was gloved for 3 h to assess the 3 h effect. The size of the surgical glove was documented for each subject and each experiment. After the surgical glove was removed, sampling was performed as for the immediate effect. A 1:10 dilution of the sampling fluid obtained from each hand was prepared in TSB. Aliquots of the sampling fluid (1 ml and 0.1 ml) and the dilution step (0.1 ml) were taken and spread onto TSA dishes with a sterile glass spatula. The dishes were incubated at 36 ± 1°C for 48 h and the CFU counted when there were between 15 and 300 colonies per plate.

The mean of CFU was calculated for each dilution. The mean was then multiplied by the dilution factor to obtain the number of CFU per ml of the sampling liquid. All pre- and post-values were expressed as log_10_ values. For calculation purposes, values of 0 (log 0 = –∞) were reset to 1 (log 1 = 0). If values in the range that could be used in calculations were obtained from more than one dilution, their mean was used as the final logarithmic value. For each volunteer, the log_10_ reduction was calculated as the difference between the log_10_ pre-value and log_10_ post-values.

Statistical analysis of multiple means was performed with ANOVA, including Tukey’s post hoc test. Repeated measurements were not accommodated in the statistical analysis. A linear regression analysis was used to determine the impact of glove size and volume of disinfectant applied on the overall efficacy. All calculations were performed using PASW Statistics 18, SPSS Inc., Chicago, USA. A p-value < 0.05 was considered significant.

## Results

A total of 13 experiments were performed according to EN 12791, resulting in 269 surgical reference disinfections in 75 different subjects. Forty-six subjects participated 1–3 times (61.3%), 14 subjects 4–6 times (18.7%), seven subjects 7–9 times (9.3%), and eight subjects 10–13 times (10.7%). Similar proportions of repeated measurements per subject were found for all glove sizes (Table 1). Most data were obtained from subjects with a glove size of 7.5 (n = 106), followed by a glove size of 8 (n = 68), a glove size of 7 (n = 62), a glove size of 8.5 (n = 24), and a glove size of 9 (n = 9). Most surgical hand disinfection procedures were performed with 9 ml of alcohol (n = 185), followed by 12 ml (n = 45) and 6 ml (n = 39). The mean pre-value for resident hand bacteria before application of the reference alcohol was 4.41 ± 0.79 log_10_ CFU. There was no significant difference between the mean pre-values for the different hand sizes (p = 0.540, ANOVA; range of means: 4.25 ± 0.95 for glove size 9, 4.67 ± 0.66 for glove size 8.5).

**Table 1:** Number of subjects per glove size and number of participations

**Glove size**	**1 - 3 participations**	**4 - 6 participations**	**7 - 9 participations**	**10 - 13 participations**
7	14	3	2	1
7.5	19	6	2	4
8	8	1	2	3
8.5	5	2	1	0
9	0	2	0	0

The mean immediate efficacy of all 269 surgical hand disinfection procedures was 2.56 ± 1.12. The mean log_10_ reduction was somewhat higher in subjects with glove sizes 7 (2.83 ± 1.02) and 8.5 (2.62 ± 1.12), but the difference between all glove sizes was not significant (p = 0.182, ANOVA; Table 2). The application of 6 ml of the reference alcohol produced the lowest mean log_10_ reduction for all glove sizes, e.g., 1.76 ± 1.18 in subjects of glove size 7.5 and 1.79 ± 1.22 in subjects of glove size 8. The difference between the three volumes was only significant in subjects with glove size 7.5 (p = 0.012; ANOVA). Although there was no significant difference between the efficacy of all three volumes in subjects with glove size 8 (p = 0.05), the post hoc analysis revealed that the application of 6 ml was significantly less effective than the application of 9 ml (p = 0.043, Tukey). A linear regression analysis revealed that a larger volume correlated significantly with a better immediate efficacy (T = 2.782, p = 0.006), whereas a smaller glove size did not (T = –1.337, p = 0.182).

**Table 2:** **Immediate log**_**10 **_**reduction of 60% n-propanol for surgical hand disinfection applied for 3 min according to EN 12791 in 269 subjects**

**Size of glove**	**Mean log**_**10**_**-reduction (number of subjects)**	**6 ml (number of subjects)**	**9 ml (number of subjects)**	**12 ml (number of subjects)**	**p-value**
7	2.83 ± 1.02 (62)	2.30 ± 1.05 (11)	2.88 ± 0.94 (43)	3.27 ± 1.28 (8)	0.106
7.5	2.52 ± 1.08 (106)	1.76 ± 1.18 (15)*	2.66 ± 1.03 (77)*	2.56 ± 0.96 (14)	0.012
8	2.48 ± 1.23 (68)	1.79 ± 1.22 (13)*	2.71 ± 1.13 (45)*	2.32 ± 1.43 (10)	0.05
8.5	2.62 ± 1.12 (24)	-	2.77 ± 1.25 (17)	2.25 ± 0.67 (7)	0.311
9	2.03 ± 1.04 (9)	-	1.92 ± 0.27 (3)	2.09 ± 1.30 (6)	0.828

*post hoc analysis using Tukey < 0.05.

The mean 3 h efficacy of all 269 surgical hand disinfection procedures was 2.12 ± 1.24. The mean log_10_ reduction was somewhat lower in subjects with glove size 9 (1.03 ± 1.24), but the difference between all glove sizes was not significant (p = 0.051, ANOVA; Table 3). Similar to the data for the immediate efficacy, the application of 6 ml of the reference alcohol produced the lowest mean log_10_ reduction in all glove sizes, e.g., 1.27 ± 1.19 in subjects with glove size 8 and 1.32 ± 0.97 in subjects with glove size 7.5. The difference between the three volumes was significant in subjects of glove size 7 (p = 0.021; ANOVA), glove size 7.5 (p = 0.025), and glove size 8 (p = 0.013). The post hoc analysis revealed that the application of 6 ml was significantly less effective than the application of 9 ml (glove sizes 7.5–8) or the application of 12 ml (glove size 7; p < 0.05, Tukey). A linear regression analysis revealed that both a larger volume (T = 2.650, p = 0.009) and a smaller glove size (T = –2.064, p = 0.040) correlated significantly with better 3 h efficacy.

**Table 3:** **Mean log**_**10 **_**reduction after 3 hours of 60% n-propanol for surgical hand disinfection applied for 3 min according to EN 12791 in 269 subjects**

**Size of glove**	**Mean log**_**10**_**-reduction (number of subjects)**	**6 ml (number of subjects)**	**9 ml (number of subjects)**	**12 ml (number of subjects)**	**p-value**
7	2.25 ± 1.20 (62)	1.73 ± 0.86 (11)*	2.20 ± 1.17 (43)	3.23 ± 1.31 (8)*	0.021
7.5	2.03 ± 1.11 (106)	1.32 ± 0.97 (15)*	2.14 ± 1.12 (77)*	2.21 ± 0.97 (14)	0.025
8	2.18 ± 1.32 (68)	1.27 ± 1.19 (13)*	2.47 ± 1.33 (45)*	2.10 ± 0.92 (10)	0.013
8.5	2.37 ± 1.49 (24)	-	2.51 ± 1.50 (17)	2.05 ± 1.53 (7)	0.502
9	1.03 ± 1.24 (9)	-	1.50 ± 0.70 (3)	0.79 ± 1.43 (6)	0.461

*post hoc analysis using Tukey < 0.05.

## Discussion

The volume of disinfectant applied seems to be a major factor in achieving the optimum efficacy in surgical hand disinfection. Our data indicate that in subjects with glove sizes of 7–8, 6 ml of n-propanol (60%) was less effective than 9–12 ml both immediately and after 3 h. Subjects with a glove size of 8.5 or 9 always used 9 ml or more to keep their hands wet with the reference alcohol for 3 min, indicating that 6 ml is insufficient for an application time of 3 min on large hands. A volume of 6 ml has been described as effective for surgical hand disinfection with a propanol-based hand rub and an application time of 1.5 min when only the hands are treated [6]. The treatment of hands and forearms has been described to be equally effective in 1.5 min [13], but will certainly require a larger volume of disinfectant for the same application time. Therefore, it is unlikely that small volumes, such as 6 ml or even 4 ml, are sufficient for the entire surgical hand disinfection procedure in subjects with large hands, especially when the volume must be sufficient for both hands and forearms or lower forearms.

Hand size, which was measured as glove size, appeared to be a minor factor in achieving optimal efficacy in surgical hand disinfection. When we assessed the immediate efficacy, glove size had no significant effect on efficacy, whereas after 3 h, glove size had a barely significant effect. The smallest glove size in our subjects was 7, the largest glove size was 9. Manufacturers of surgical gloves also offer gloves smaller than size 7, beginning at a size of 5.5, whereas gloves larger than size 9 are not routinely available. Therefore, our findings probably cover the large hands commonly present in surgical theaters.

A limitation of our study is that we used the reference procedure for surgical hand disinfection described in EN 12791, which requires the application of 60% (v/v) n-propanol for 3 min. We have no data showing that commercially available hand rubs produce equivalent results. The products currently recommended to be used in quite small volumes (4 ml or 6 ml) contain ethanol as the main or only active ingredient, at a concentration of 62% or 70%. It has previously been reported that the type of alcohol used is the most important factor in the optimum disinfection of the resident skin flora, and that n-propanol is more effective than ethanol or isopropanol [14]. Therefore, we expect that these formulations would produce similar results to those observed in this study.

Another limitation is that we used existing data sets, obtained according to the EN 12791 protocol, with variable numbers of subjects with specific glove sizes, resulting in a quite small number of subjects with a glove size of 9 or 8.5, and few corresponding data. The same number of subjects in each subgroup with a specific glove size would have been preferable, but nevertheless, we consider that our data demonstrate a phenomenon of clinical relevance. The clinical impact of using an insufficient volume of hand rub for surgical hand disinfection has been reported recently in an outbreak of surgical-site infections [15]. This clinical correlation with our results clearly supports our findings.

Based on our data, the current continuous reduction in the volume of surgical hand preparation applied is critical, especially when large hand sizes are not taken into account. If the efficacy of a small volume of a formulation is demonstrated in subjects with small or medium hands, it will probably be significantly less effective in subjects with large hands. Therefore, it should be mandatory that surgical hand products recommended for use in small volumes provide clear evidence that the formulations are equally effective in subjects with large hands. Patients may otherwise be put at risk, because contaminated glove juice has been shown to increase the risk of surgical site infections [16].

## Competing interests

Both authors are employed by Bode Chemie GmbH, Hamburg, Germany.

## Authors’ contributions

GK designed the study, analyzed the data, and drafted the manuscript. CO contributed the data, revised the manuscript, and gave final approval. Both authors read and approved the final manuscript.
